# Participant referral rate in the National Eye Health Survey (NEHS)

**DOI:** 10.1371/journal.pone.0174867

**Published:** 2017-04-04

**Authors:** Stuart Keel, Pei Ying Lee, Joshua Foreman, Peter van Wijngaarden, Hugh R. Taylor, Mohamed Dirani

**Affiliations:** 1 Centre for Eye Research Australia, University of Melbourne, Melbourne, Victoria, Australia; 2 Department of Ophthalmology, University of Melbourne, Melbourne, Victoria, Australia; 3 Indigenous Eye Health Unit, Melbourne School of Population and Global Health, The University of Melbourne, Melbourne, Victoria, Australia; Swinburne University of Technology, AUSTRALIA

## Abstract

**Purpose:**

To present the rates of referral of participants in the National Eye Health Survey (NEHS) for further eye care.

**Materials & methods:**

A national sample of 3098 non-Indigenous Australians aged 50–98 and 1738 Indigenous Australians aged 40–92 years living in 30 randomly selected sites was recruited using a door-to-door approach. Participants completed a general questionnaire and a series of eye tests, including vision and anterior segment assessment, intra-ocular pressure measurement, visual field testing and fundus photography. A predefined protocol was used to guide the referral of participants for follow up eye care. An ophthalmologist was on-call to assist with the triaging of participants.

**Results:**

Of the total sample, 32.1% (994/3098) of non-Indigenous participants and 43.6% (757/1738) of Indigenous participants were referred for further eye care (p<0.001). A significant difference in referral rates for Indigenous Australians was observed between regions of differing geographic remoteness [range = 32.2% (Inner Regional)—60.4% (Very Remote), p <0.001]. After adjusting for covariates, males (OR = 1.24, 95% CI: 1.06–1.46), older age (OR = 1.02 per year, 95% CI: 1.01, 1.02) and longer time since previous eye examination (OR = 1.15 per year, 95% CI: 1.12, 1.19) were associated with higher rates of eye care referral in the non-Indigenous population. In the Indigenous population, older age (OR = 1.02 per year, 95% CI: 1.01–1.03), self-reported diabetes (OR = 1.70, 95% CI: 1.37–2.12), greater geographical remoteness (OR = 1.19, 95% CI: 1.09–1.29) and longer time since previous eye examination (OR = 1.10 per year, 95% CI: 1.07, 1.13) were associated with a higher rate of referral after multivariate adjustments. A total of 25 participants (1.4%) were referred for urgent follow-up of potentially sight threatening conditions.

**Conclusions:**

Our data has identified several high risk groups that required ophthalmic referral including older Australians, non-Indigenous men, Indigenous Australians with self-reported diabetes and those residing in very remote populations who may benefit from improvements in the provision and/or uptake of eye health services. Future longitudinal research is warranted to evaluate the feasibility and efficacy of implementing a referral protocol within a population-based research setting.

## Introduction

In 2012 the World Health Organisation (WHO) reported that more than 300 million people worldwide were vision impaired or blind [1]. It has been estimated that 80% of vision impairment and blindness can be treated or avoided through early detection and prevention [2]. In Australia, two state-based studies (n = 8909) conducted in the early 1990s collectively estimated that 480,300 Australians had vision impairment (visual acuity <6/12), including 50,600 with blindness (visual acuity <6/60) [3]. The National Eye Health Survey (NEHS) commenced in 2015 with the aim of providing up-to-date national data on the prevalence of vision impairment and blindness in Indigenous and non-Indigenous Australians. The NEHS testing protocol included predefined referral guidelines to ensure that participants with low vision, those suspicious for eye disease and those that do not adhere to recommended guidelines for general or diabetes ocular examinations were referred to the most appropriate healthcare provider in a timely fashion.

Most population-based eye health studies either fail to include referral guidelines in their study protocols, or do not report referral rates or the reasons for referral [4–8]. Only a few studies have reported referral rates for follow up eye care and most of these relate to eye screening programs as opposed to population-based studies [9,10]. For instance, Friedman and co-workers (2012) evaluated a vision screening program for under-served populations in the United States and reported referral rates of 46% (1380/3004), while Looker and co-workers (2014) reported a referral rate of 11% (20,952/187,822) in the Scottish National Diabetic Retinopathy Screening Program. To date, only one population-based eye study conducted on homeless adults in the Canadian city of Toronto has reported rates of urgent ophthalmology referrals (8% of study participants) [11].

The implementation of a referral protocol in a research study requires adequate resources including personnel, clinical expertise and governance systems. Whilst a population health study should not be misconstrued as the provision of health screening or health care, the detection of disease or individuals at risk of disease in the course of a study should, in our view, warrant timely referral. This is particularly relevant in ophthalmic research given that the large majority of eye diseases are asymptomatic in their early stages and vision loss is often treatable or avoidable through appropriate interventions [2]. This paper describes the referral guidelines and referral rates for participants in the NEHS.

## Methods

The protocol for this study was approved by the Human Research Ethics Committee of the Royal Victorian Eye and Ear Hospital (RVEEH) (HREC-14/1199H), the Aboriginal Health and Medical Research Council of New South Wales (HREC-1079/15), the Aboriginal Health Council of Western Australia (HREC-622), the Menzies School of Health Research (HREC-2015-2360) and the Aboriginal Health Council of South Australia (HREC-04-15-604). This research was conducted in accordance with the Declaration of Helsinki.

### Sampling and recruitment

The selection of recruitment sites was based on the Australian Statistical Geography Standard (ASGS), developed by the Australian Bureau of Statistics (ABS) to report 2011 Census data. Thirty sites, across five Remoteness Areas (Major City, Inner Regional, Outer Regional, Remote and Very Remote), were selected using a multi-stage, random cluster sampling methodology. To obtain a nationally representative sample of the population, 100 non-Indigenous Australians aged 50 years and older and 50 Indigenous Australians aged 40 years and older were to be recruited at each site. Recruiters went door-to-door to determine the eligibility of the residents. All eligible residents were invited to participate. Engagement of local Aboriginal health workers and community elders played a central role in the recruitment of Indigenous participants.

### Participant questionnaire and examination methodology

Clinical examinations took place in a venue that was within 6km of the target recruitment area. The testing protocol took approximately 30 minutes per participant.

#### Consent and questionnaire

All participants provided informed, written consent prior to examination. Each participant underwent an interviewer-administered questionnaire to collect information on socio-demographic factors, history of ocular problems, stroke and diabetes. Participants then underwent a series of eye tests, administered by trained eye examiners.

#### Vision and refraction

Presenting distance visual acuity (VA) was measured using a logMAR chart (Brien Holden Vision Institute, Australia). If presenting VA was worse than 6/12 (0.3 logMar equivalent) in either eye, vision was retested with a pinhole occluder. Auto-refraction was performed using a hand-held auto-refractor/keratometer (Nidek ARK-30 Type-R, Nidek Co., LTD, Japan) to objectively measure refractive error of participants whose VA improved with pinhole to ≥6/12. Presenting near vision was assessed using the CERA E near vision card at the participant’s preferred reading distance [12].

#### Anterior segment assessment

Anterior segment assessment of both eyes was conducted using a hand-held slit lamp (Keeler PSL One, Keeler Ophthalmic Instruments, UK) at 10x magnification for the presence of pterygium and lid abnormalities. Trachoma grading was conducted using the WHO Trachoma Simplified Grading System [13] in Indigenous participants only as this condition was eradicated in non-indigenous Australians earlier this century [14]. Anterior segment photographs were taken using a non-mydriatic Diabetic Retinopathy Screening (DRS) camera (CenterVue SpA, Italy) in participants with presenting distance VA of <6/12 in one or both eyes.

#### Perimetry and Intraocular Pressure (IOP)

The N-30-5 screening protocol of the Frequency Doubling Technology (FDT) perimeter (Carl Zeiss Meditec & Welch Allyn, USA) was used to assess visual field loss. If the sensitivity was reduced in any of the field locations, the test was repeated to determine the reproducibility of the defect and the best result was graded. Intraocular pressure (IOP) was measured using a tonometer (iCare, Finland).

#### Retinal photography

Two-field, forty-five degree colour fundus photography was performed using a non-mydriatic fundus camera (DRS, CenterVue SpA, Italy) to assess the retina and the optic disc. If retinal photographs of reduced quality were due to small pupil size (<3.00mm), the pupil was dilated with tropicamide 0.5% and photographs were retaken. Anterior chamber angle depth estimation was performed with a hand-held slit lamp using the Van Herick grading system, prior to pupil dilation to exclude participants at risk of anterior chamber angle closure [15].

### Referral protocol

At the completion of the examination, each participant was provided with verbal feedback on the health of their eyes. This included a discussion of VA, IOP, visual fields as well as anterior and posterior ocular health. A referral protocol was developed by study investigators in conjunction with ophthalmologists (Table 1). Participants were provided with a referral letter to be taken to their optometrist or local doctor if they met any of the following referral criteria: (1) evidence of eye disease or visual impairment detected during the NEHS eye examination; [16] participants with diabetes who had not undergone a screening eye examination within the timeframe recommended by the National Health and Medical Research Council (NHMRC) diabetic retinopathy guidelines [17], or (3) individuals without diabetes who had undergone an eye examination in the past 5 years [18]. The referral letter provided an overview of the study, and an outline of eye examination findings including vision, visual field, and anterior and posterior segment assessment. Participants were advised of the appropriate timing for follow up, ranging from same day to 3 months, according to the nature of the findings. Participants who were already under ophthalmological care were not provided with a referral unless new pathology was suspected.

**Table 1:** Referral protocol in the NEHS.

Test	Findings	Timing of Referral
VA	Presenting VA <6/12 in either eye	1–2 months (urgency dictated by onset and severity of vision loss
		Within 1 week if VA <6/12 both eyes and participant is driving
FDT	≥2 points missed in either eye (for best result)	1–2 month
	Diabetic retinopathy	1 month (haemorrhages & exudates, if eye health care provider has not been seen in the last 3 months)
		1 week (central exudates & reduced vision—macular oedema, or any proliferative retinopathy)
		**NB**: *Annual examinations for Indigenous persons and at least every 2 years for non-Indigenous Australians with diabetes*
	Age related macular degeneration	1 month (large drusen, pigment change or atrophy)
		1–2 days (any sub-retinal blood in macula, or new symptoms of distortion, scotoma, vision loss)
	Glaucoma suspect (C:D ≥0.4, asymmetry >0.2 C:D, peripapillary atrophy, retinal nerve fiber layer defect)	1 month, unless under care
	Pigmented lesion (choroidal naevus or melanoma)	1 month (naevus), 1 week (melanoma)
	Vitreous haemorrhage, retinal vascular occlusion, retinal tear or detachment	Same day
Trachoma grading	Trachomatous trichiasis, corneal opacity	1 month
Unilateral red eye	Especially if acute and painful; photophobic	Same day
IOP	IOP>21mmHg (non-urgent)	2 weeks (sooner if C:D >0.8)
	IOP>30mmHg (urgent)^1^	Same day
Van Herrick	≤ Grade 2 in either eye	1 month (check for symptoms of angle closure glaucoma—urgent referral if so)
	Symptoms:	
	• Flashing lights (persistent, recent onset), transient visual obscuration (amaurosis)^1^	Same day
	• Recent headaches (if severe; or temporal ache)	1–2 days
	• Red colour desaturation, photophobia (marked light sensitivity), recent history of significant eye trauma	1–2 days
	Signs:	
	• Reddening of the peri-ocular skin (cellulitis)	Same day (**Urgent** if double vision; reduced motility or proptosis)
	• Corneal ulcers or opacification	Same day
	• Lid lesions	1 week (if raised, irregular lid margin & vascularised), regular review (if longstanding)
	• Pterygium encroaching visual axis	Same timing as VA referral
	• Significant cataract	1 month
	• Conjunctival lesions	1 week (if raised & vascularised), regular review (if longstanding)
	Other issues on history:	
	• Using unspecified eye drops/naturopathy eye drops etc., strong family history of eye disease	1 month

^1^A phone referral was made if acute glaucoma or giant cell arteritis were suspected

Referrals were made by optometrists, orthoptists or medical doctors within the research team. A consultant ophthalmologist was available at all times via telephone to provide advice on clinical findings and to triage referrals when necessary. Records of referrals for Indigenous participants were provided to the corresponding local Aboriginal health services with the consent of the participants.

### Statistical analysis

Statistical analyses were performed with Statistical Package for the Social Sciences (SPSS) (version 24; Chicago, IL, USA). A p-value of 0.05 was used for significance testing. Descriptive statistics were calculated and Kolmogorov-Smirnov or Shapiro-Wilk significance statistic was used to assess the normality of the distribution of scores with a non-significant result (p = ≥0.05) indicating normality. Chi-square tests were utilised to compare proportions and an independent-samples t-test was used to compare continuous variables of interest between; 1) referred and non-referred participants, 2) referred Indigenous and referred non-Indigenous participants. Chi-square tests were utilised to compare referral rates between the 5 Remoteness Areas for Indigenous and non-Indigenous populations. Multivariable logistic regression analysis was used to examine the association between referral status and all covariates that were significant in univariate analysis.

## Results

### Key demographics of participants who received a referral in the NEHS

A total of 4836 individuals were recruited and examined in the NEHS, including 3098 (64.1%) and 1738 (35.9%) non-Indigenous and Indigenous Australians, respectively. Almost one-third (994/3098, 32.1%) of non-Indigenous participants (mean [SD] age of 67.0 [10.0] years, range 50 to 98 years) in the study were referred for further eye examination and management. In contrast 43.6% (757/1738) of Indigenous participants (mean [SD] age of 56.1 [10.5] years, range 40 to 92 years) were referred (p<0.001) (Table 2). A significant difference in referral rates for Indigenous Australians was observed between regions of differing geographic remoteness (range = 32.2% (Inner Regional)—60.4% (Very Remote), p <0.001) (Table 3).

**Table 2:** Sociodemographic characteristics of participants who received ophthalmic referral and those who did not, stratified by Indigenous status.

	Non-Indigenous (n = 3098)		Indigenous (n = 1738)	
	Referred (n = 994)	Not referred (n = 2104)	Referred (n = 757)	Not referred (n = 981)
Mean age (SD)	67.0 (10.0)	66.4 (9.5)	56.1 (10.5)	54.2 (9.5)
Mean years of education (SD)	12.3 (3.8)	12.7 (3.7)	10.7 (3.4)	11.2 (3.3)
Gender (n, % male)	515 (51.8)	922 (43.8)	323 (42.7)	391 (39.9)
Self-reported stroke (n, %)	53 (5.3)	103 (7.3)	91 (12.0)	61 (6.2)
Self-reported diabetes (n, %)	127 (12.8)	304 (14.4)	327 (43.2)	318 (32.4)

**Table 3:** Proportion of non-Indigenous and Indigenous participants referred by Remoteness Area.

	Non-Indigenous			Indigenous		
	N	n^1^, (%)	p value	N	n^1^, (%)	p value*
Major City	1253	383 (30.6)	0.13	746	292 (39.1)	<0.001
Inner Regional	636	192 (30.2)		310	103 (33.2)	
Outer Regional	625	219 (35.0)		405	214 (52.8)	
Remote	367	131 (35.7)		181	90 (49.7)	
Very Remote	217	69 (31.8)		96	58 (60.4)	

*p values are based on the chi-squared test comparing the proportion of participants referred between Remoteness Areas.

^1^Number of participants who were referred.

### Associations between eye care referral and selected characteristics

In the non-Indigenous population, adjusted analysis revealed that males (OR = 1.24, 95% CI: 1.06–1.46), older age (OR = 1.02, 95% CI: 1.01, 1.02) and longer time since previous eye examination (OR = 1.15 per year, 95% CI: 1.12, 1.19) were associated with higher rates of eye care referral. For Indigenous Australians, older age (OR = 1.02, 95% CI: 1.01–1.03), self-reported diabetes (OR = 1.70, 95% CI: 1.37–2.12), greater geographical remoteness (OR = 1.19, 95% CI: 1.09–1.29) and longer time since previous eye examination (OR = 1.10 per year, 95% CI: 1.07, 1.13) were associated with higher rates of eye care referral after multivariate adjustments (Table 4).

**Table 4:** Univariate and multivariable logistic regression analysis of associations between participant referral and selected characteristics in A) non-Indigenous participants and B) Indigenous participants.

	A) Non-Indigenous (n = 3,098)				B) Indigenous (n = 1,738)			
Associated factors	Univariate		Multivariate^1^		Univariate		Multivariate^1^	
	Unadjusted OR (95% CI)	P*	Adjusted OR (95% CI)	P	Unadjusted OR (95% CI)	P	Adjusted OR (95% CI)	P
**Non-Indigenous**								
Age (year)	1.01 (0.99, 1.02)	0.08	1.02 (1.01, 1.02)	<0.001	1.02 (1.01, 1.03)	<0.001	1.02 (1.01, 1.03)	0.001
Gender (male)	1.38 (1.19, 1.60)	<0.001	1.24 (1.06, 1.46)	0.007	1.12 (0.93, 1.36)	0.02	1.06 (0.85, 1.31)	0.62
Education (year)	0.98 (0.96, 1.00)	0.02	0.98 (0.96, 1.00)	0.11	0.96 (0.93, 0.99)	0.005	0.97 (0.94, 1.00)	0.10
Diabetes (self-report)	0.87 (0.69, 1.08)	0.21	0.83 (0.67, 0.04)	0.11	1.59 (1.30, 1.93)	<0.001	1.70 (1.37, 2.12)	<0.001
Stroke (self-report)	1.00 (0.99, 1.00)	0.76	1.00 (0.99, 1.00)	0.64	1.00 (0.99, 1.00)	0.60	1.00 (0.99, 1.00)	0.91
Remoteness level	1.06 (1.00, 1.12)	0.06	1.04 (0.97, 1.10)	0.27	1.24 (1.15, 1.34)	<0.001	1.19 (1.09, 1.29)	<0.001
Time since last exam (year)	1.15 (1.12, 1.19)	<0.001	1.15 (1.12, 1.19)	<0.001	1.08 (1.05, 1.11)	<0.001	1.10 (1.07, 1.13)	<0.001

OR = Odds Ratio, CI = Confidence Interval

*Statistical significance was set as a p value of ≤0.05 (two tailed).

^1^Model adjusted for: age, gender, years of education, remoteness, time since last eye exam, history of diabetes and history of stroke.

### Timing of referrals

Over three quarters of referrals for non-Indigenous (784/994, 78.9%) and Indigenous (573/757, 75.7%) participants were recommended for one month (Table 5), while a total of 25 participants (1.4%) were referred for urgent follow-up (same or next day) to local optometrists, ophthalmologists or hospital emergency departments.

**Table 5:** Recommended timing of referral for non-Indigenous and Indigenous participants.

	n, %	
	Non-Indigenous	Indigenous
Urgent—same/next day	13 (1.3)	12 (1.6)
1 week	83 (8.4)	70 (9.3)
2 weeks	100 (10.1)	74 (9.8)
1 month	784 (78.9)	573 (75.7)
2 or more months	14 (1.4)	28 (3.7)

### Reasons for referrals

#### Overview

The reasons for referral were grouped into 5 categories: repeatable visual field loss, high IOP (>21mmHg), anterior segment abnormality, posterior segment abnormality and non-adherence to recommended guidelines for general or diabetes ocular examinations (Table 6). The three leading reasons for referrals in non-Indigenous participants were macular drusen or pigment changes (206/994, 20.7%), findings suggestive of glaucoma (203/994, 20.2%) and non-adherence to general or diabetic ocular examinations (198/994, 19.9%). Non-adherence to general or diabetic ocular examinations (403/757, 53.2%,), signs of glaucoma (149/757, 19.7%) and findings suggestive of diabetic retinopathy (136/757, 18.0%) were primary reasons for referral for Indigenous participants. 14.4% of referrals for non-Indigenous participants and 18.3% of referrals for Indigenous participants were made based on a presenting visual acuity of less than 6/12 in one or both eyes due to suspected cataract or refractive error. Of note, an assessment of the best-corrected visual acuity (BCVA) in the better eye of the 1751 persons referred revealed that 1660 (94.8%) individuals had a BCVA that was 6/12 or better, 88 (5.0%) had a BCVA between <6/12-6/60 and 3 (0.2%) had a BCVA less than <6/60 in their better eye.

**Table 6:** Reasons for referral, stratified by Indigenous status.

Reason	Non-Indigenous (n, %)	Indigenous (n, %)
***Posterior segment***		
Choroidal nevus	23 (2.3)	6 (0.79)
Diabetic retinal signs	38 (3.8)	136 (18.0)
Epiretinal membrane	58 (5.8)	13 (1.7)
Glaucoma suspect^1^	203 (20.4)	149 (19.7)
Hypertensive retinal changes	48 (4.8)	38 (5.0)
Macular drusen or pigment changes	206 (20.7)	101 (13.3)
Other^2^	40 (4.0)	9 (1.2)
***Anterior segment***		
Cataract/Refractive error^3^	143 (14.4)	139 (18.3)
Lid lesion	7 (0.70)	7 (0.92)
Narrow anterior chamber angles (≤ grade 2)	16 (1.6)	14 (1.9)
Pterygium encroaching visual axis	6 (0.60)	12 (1.6)
Other^4^	8 (0.08)	17 (2.3)
***Miscellaneous***		
High intraocular pressure (>21mmHg)	106 (10.7)	53 (7.0)
Non-adherence to general or diabetic ocular examination guidelines^5^	198 (19.9)	403 (53.2)
Visual field defect (≥ 2 points missed)	170 (17.1)	130 (17.2)

^1^Glaucoma suspect = cup:disc ≥0.4, asymmetry >0.2 cup:disc, peripapillary atrophy, retinal nerve fiber layer defect.

^2^Other posterior segment reasons = retinal scar, asteroid hyalosis, posterior vitreous detachment with photopsia, optic nerve head coloboma, central serous retinopathy or multiple evanescent white dot syndrome

^3^Cataract or refractive error resulting in a presenting visual acuity of <6/12 in one or both eyes

^4^Other anterior segment reasons = blepharitis, dry eye, trichiasis, conjunctival lesion or keratoconus

^5^Current NHMRC guidelines recommend a diabetic eye examination annually for Aboriginal or Torres Strait Islander persons with diabetes and at least every 2 years for non-Indigenous Australians with diabetes. Current guidelines for general eye examinations in individuals without diabetes is at least every 5 years.[18]

#### Reasons for urgent referrals

Of the 25 participants referred for urgent follow-up, 12 (48%) presented with an IOP of >30mmHg, 10 (40%) presented with symptoms consistent with possible retinal tears (persistent photopsias of recent onset), 2 (8%) showed signs of uveitis, and 1 (4%) participant had presumed periorbital cellulitis.

#### Primary reasons for semi-urgent (1 week) referrals

Of those participants who required semi-urgent (1 week) referrals (n = 153), the five primary reasons for referral were: presenting vision less than the Australian driving standard (6/12) in both eyes (n = 81, 41.5%); a recent onset of unilateral vision loss (VA<6/60) (n = 13, 7.3%); retinal hemorrhage (n = 12; 6.7%), and elevated IOP (≥21mmHg) with an enlarged vertical optic cup to optic disc ratio (≥0.8) (n = 8; 4.5%).

## Discussion

The NEHS is a population-based study conducted between March 2015 and April 2016 that aimed to determine the prevalence of vision impairment and blindness in Australia. Herein we have described the rate of referral of NEHS participants for further eye care and identified risk factors for referral.

The overall referral rate for the study was high, with more than 1 in 3 participants being directed to a local eye health or primary care service for follow-up assessment. The significance of this finding must not be overstated as the vast majority of referrals were due to non-urgent reasons, such as poor compliance to recommended guidelines for general or diabetic ocular examinations. Of note however, approximately 10% (178/1738) of the referrals made by NEHS examiners recommended urgent (same day/next day) or semi-urgent (within one week) follow-up, accounting for 3.9% of the total sample. Urgent and semi-urgent referrals represent undetected and potentially sight-threatening ocular conditions that were opportunistically detected. It can be speculated that had these individuals not participated in the NEHS their ocular disease may have gone undetected, particularly for those who had never had an eye examination. It is not easy to put these referral rates into context given the paucity of similar data from other population-based eye studies. The urgent referral rate in the current study (1.4%) was lower than that reported amongst homeless adults in the Canadian city of Toronto of 8% [9]. These results are difficult to compare due to the vast differences in the sample populations and the referral protocols employed.

With the exception of non-adherence to recommended guidelines for general or diabetic ocular examinations, the main reason for referral of participants was the finding of suspicious retinal or optic nerve pathology. Population studies have demonstrated that the prevalence of asymptomatic retinal abnormalities increases with age [19]. While the clinical outcomes of the NEHS participants who were referred for follow-up are not known, it is apparent that population eye health studies may contribute to the opportunistic detection of disease and, when coupled with a clinical referral protocol, could promote improved eye health outcomes for participants.

Indigenous Australian participants (757/1738, 43.6%) in the NEHS were more likely than non-Indigenous Australian participants (994/3098, 32.1%) to be referred for follow-up and this was the case for all Remoteness Areas studied. It appears that this finding can be largely explained by a higher prevalence of self-reported diabetes (Indigenous = 37.1% vs. non-Indigenous = 13.9%) coupled with poorer adherence to recommended guidelines for diabetes ocular examinations in Indigenous Australians. A lack of awareness of the more stringent recommendation for annual diabetic retinal screening for Indigenous Australians, as opposed to biennial screening for most non-Indigenous Australians, may account for the sub-optimal compliance to these guidelines [17]. This is consistent with previous studies that have identified a low uptake of eye examinations by Indigenous Australians with diabetes [20–22]. Individuals with diabetes who do not have their eyes examined at the recommended frequency place themselves at risk of diabetic retinopathy. Therefore, the finding in Indigenous participants of a higher rate of referral due to diabetic retinopathy is not surprising. In recent times, improvement in the uptake of services by Indigenous Australians has been a focus of the Australian Government, with the introduction of new Medicare Benefits Schedule [23] items for primary care services to cover diabetic retinal screening using non-mydriatic retinal photography [24]. Our findings may provide support for this initiative.

Referral rates differed significantly for Indigenous study participants by Remoteness Areas (range = 39.1%–60.4%) but not for non-Indigenous participants (range = 30.2%–35.7%). Notably, referral rates for Indigenous participants in very remote sites were approximately 1.5 to 2 times higher than those of Major City and Inner Regional sites. While referral rates may not directly correlate with the burden of eye disease within communities, these findings may be a reflection of the well-established barriers that Indigenous Australians face in accessing specialist services in remote areas, including communication, distance, and cultural inappropriateness [25,26]. To compound this, a recent review has identified a disproportionately low availability of services in remote Australia, with the number of patients per optometrist being 12,700 in remote areas compared with the national average of approximately 1,180 [27,28]. Numerous eye health care agencies are currently undertaking major efforts to improve Indigenous eye health through better access to education, improved funding and the implementation of specialist outreach services in many regional and remote Indigenous communities [29].

A strength of the NEHS is its representativeness, achieved through the use of population-based sampling stratified by remoteness. The use of a predefined clinical referral protocol by qualified eye health professionals has allowed the study to provide useful insights into the prevalence of referable eye disorders and vision impairment. Nevertheless, the results of the current study must be considered in light of two key limitations. Firstly, the clinical referral protocol implemented in this study has not been validated and due to time and logistical constraints it was beyond the scope of the study to obtain access to the outcomes of follow-up for referred participants. This precludes appraisal of the appropriateness of the referral criteria, including false-positive referral rates. The social costs [30] and individual psychological impact [31] of false positive referrals have been well documented. Furthermore, an absence of follow-up data precludes our ability to assess compliance rates to referral recommendations. Given that previous research indicates that less than half of those referred from eye screening services actually present to a follow-up appointment [9] the health impact of the referral protocol is likely to be sub-optimal. These factors limit our ability to draw conclusions regarding the efficacy and feasibility of the referral protocol detailed in the current study. Secondly, the detection of referable pathology and the recommended timeframe for referrals is likely to have varied somewhat between examiners. To mitigate these effects, examiners were well trained and had demonstrated knowledge of the referral guidelines. Furthermore, an on-call ophthalmologist with access to de-identified retinal images, was available to adjudicate ambiguous cases. Emphasis was placed on participant safety and in circumstances in which examiners were uncertain of the need for, or the urgency of referral, examiners erred on the side of caution.

Our data has identified several high risk groups requiring ophthalmic referral who may benefit from improvements in the provision and/or uptake of eye health services, including; older Australians, non-Indigenous males, Indigenous Australians with self-reported diabetes and those residing in very remote communities. Whilst we are not in a position to assess the impact of the referrals that were made on the eye health of participants, we hope that participants were assisted by this approach. Future longitudinal research is warranted to further evaluate the feasibility and efficacy of implementing a referral protocol within a population-based research setting.
